# Vitamin C attenuates predisposition to high-fat diet-induced metabolic dysregulation in GLUT10-deficient mouse model

**DOI:** 10.1186/s12263-022-00713-y

**Published:** 2022-07-16

**Authors:** Chung-Lin Jiang, Chang-Yu Tsao, Yi-Ching Lee

**Affiliations:** Institute of Cellular and Organismic Biology, Academia Sinica, Taipei, Taiwan

**Keywords:** Vitamin C, Type 2 diabetes mellitus, High-fat diet, Genetic predisposition, White adipose tissue

## Abstract

**Background:**

The development of type 2 diabetes mellitus (T2DM) is highly influenced by complex interactions between genetic and environmental (dietary and lifestyle) factors. While vitamin C (ascorbic acid, AA) has been suggested as a complementary nutritional treatment for T2DM, evidence for the significance and beneficial effects of AA in T2DM is thus far inconclusive. We suspect that clinical studies on the topic might need to account for combination of genetic and dietary factors that could influence AA effects on metabolism. In this study, we tested this general idea using a mouse model with genetic predisposition to diet-induced metabolic dysfunction. In particular, we utilized mice carrying a human orthologous *GLUT10*^*G128E*^ variant (*GLUT10*^*G128E*^ mice), which are highly sensitive to high-fat diet (HFD)-induced metabolic dysregulation. The genetic variant has high relevance to human populations, as genetic polymorphisms in glucose transporter 10 (GLUT10) are associated with a T2DM intermediate phenotype in nondiabetic population.

**Results:**

We investigated the impacts of AA supplementation on metabolism in wild-type (WT) mice and *GLUT10*^*G128E*^ mice fed with a normal diet or HFD. Overall, the beneficial effects of AA on metabolism were greater in HFD-fed *GLUT10*^*G128E*^ mice than in HFD-fed WT mice. At early postnatal stages, AA improved the development of compromised epididymal white adipose tissue (eWAT) in *GLUT10*^*G128E*^ mice. In adult animals, AA supplementation attenuated the predisposition of *GLUT10*^*G128E*^ mice to HFD-triggered eWAT inflammation, adipokine dysregulation, ectopic fatty acid accumulation, metabolic dysregulation, and body weight gain, as compared with WT mice.

**Conclusions:**

Taken together, our findings suggest that AA has greater beneficial effects on metabolism in HFD-fed *GLUT10*^*G128E*^ mice than HFD-fed WT mice. As such, AA plays an important role in supporting eWAT development and attenuating HFD-induced metabolic dysregulation in *GLUT10*^*G128E*^ mice. Our results suggest that proper WAT development is essential for metabolic regulation later in life. Furthermore, when considering the usage of AA as a complementary nutrition for prevention and treatment of T2DM, individual differences in genetics and dietary patterns should be taken into account.

**Supplementary Information:**

The online version contains supplementary material available at 10.1186/s12263-022-00713-y.

## Background

Type 2 diabetes mellitus (T2DM) is a major medical problem worldwide, and its development is highly affected by complex interactions between genetic and environmental (dietary and lifestyle) factors [1]. While genetic factors have been associated with T2DM in population studies [2], the effect sizes of identified variants are typically very small. In addition to their marginal effects, many genetic factors are thought to alter susceptibility by environmental factors [3], such as high-fat diet (HFD) which accelerates the development of T2DM. Therefore, gaining a better understanding of gene and environment interactions will be important for improving assessments of disease susceptibility and progression, as well as strategies for prevention and treatment. However, the identification of particular interactions between genes and the environment remains a major challenge [4].

Antioxidants, such as vitamin C (ascorbic acid, AA), have been proposed to prevent the T2DM at least partially by attenuating white adipose tissue (WAT) inflammation [5]. WAT actively regulates whole-body energy homeostasis by storing lipids and secreting adipokines [6], and HFD-induced obesity is typically associated with increased fat deposition in WAT, induction of WAT inflammation, dysregulation of adipokines, ectopic fat accumulation, and finally T2DM [7]. However, clinical trials examining the effects of AA on T2DM have thus far been inconclusive [8, 9]. We suspect that beneficial effects of AA on metabolism might be influenced by combinations of genetic and environmental factors. Such complex interactions between various genetic risk factor combinations and specific diets or lifestyle characteristics make clinical studies on the effects of AA on T2DM difficult to design. Therefore, we addressed this issue using a mouse model that is genetically predisposed to HFD-induced metabolic dysregulation. With these mice, we sought to determine whether AA effects on metabolism might be co-regulated by a particular genetic variant and diet.

We have previously shown that *GLUT10*^*G128E*^ mice are highly sensitive to HFD-induced T2DM [10]. Loss-of-function mutations in glucose transporter 10 (GLUT10) gene (*SLC2A10*) lead to a rare autosomal recessive connective tissue disorder called arterial tortuosity syndrome (ATS; OMIM 208,050) [11]. We and others demonstrated that GLUT10 transports the oxidized form of AA (dehydroascorbic acid, DHA) and regulates intracellular AA status in fibroblasts, aortic smooth muscle cells (ASMCs), and adipocytes in which GLUT10 is highly expressed [10, 12–15]. Interestingly, the chromosomal region around the *SLC2A10* locus has been associated with T2DM in sib-pair studies [16, 17]; however, the gene was not directly associated with T2DM in genome-wide association studies [10, 18–23]. Along these lines, we demonstrated that the *SLC2A10* locus is associated with T2DM intermediate phenotypes in nondiabetic human subjects [10]. We also studied the effects of GLUT10 genetic variants on metabolism using a mouse model carrying a rare human genetic variant of *SLC2A10* (*GLUT10*^*G128E*^ mice); the study revealed that *GLUT10*^*G128E*^ mice have impaired WAT development and are highly sensitive to HFD-induced obesity and metabolic dysregulation compared with WT mice [10]. As GLUT10 deficiency impairs the ability to maintain AA homeostasis but mice can synthesize AA endogenously [24], we suspected that *GLUT10*^*G128E*^ mice might require supplementation of AA to sustain normal physiological function and metabolism when fed with a HFD.

To test whether AA effects on metabolism might be influenced by combinations of genetic and environmental factors, we determined whether AA supplementation might differentially affect metabolism in wild-type (WT) mice and *GLUT10*^*G128E*^ mice fed with a normal diet (CD) or HFD. We found that AA supplementation differentially affects metabolism depending on the genetic variant and diet. Our study therefore provides strong support for the idea that clinical studies on the effects of AA in T2DM prevention should account for interactions between diet and specific genetic variants.

## Results

### AA supplementation attenuates HFD-induced metabolic dysregulation in *GLUT10*^*G128E*^ mice

**Fig. 1:**
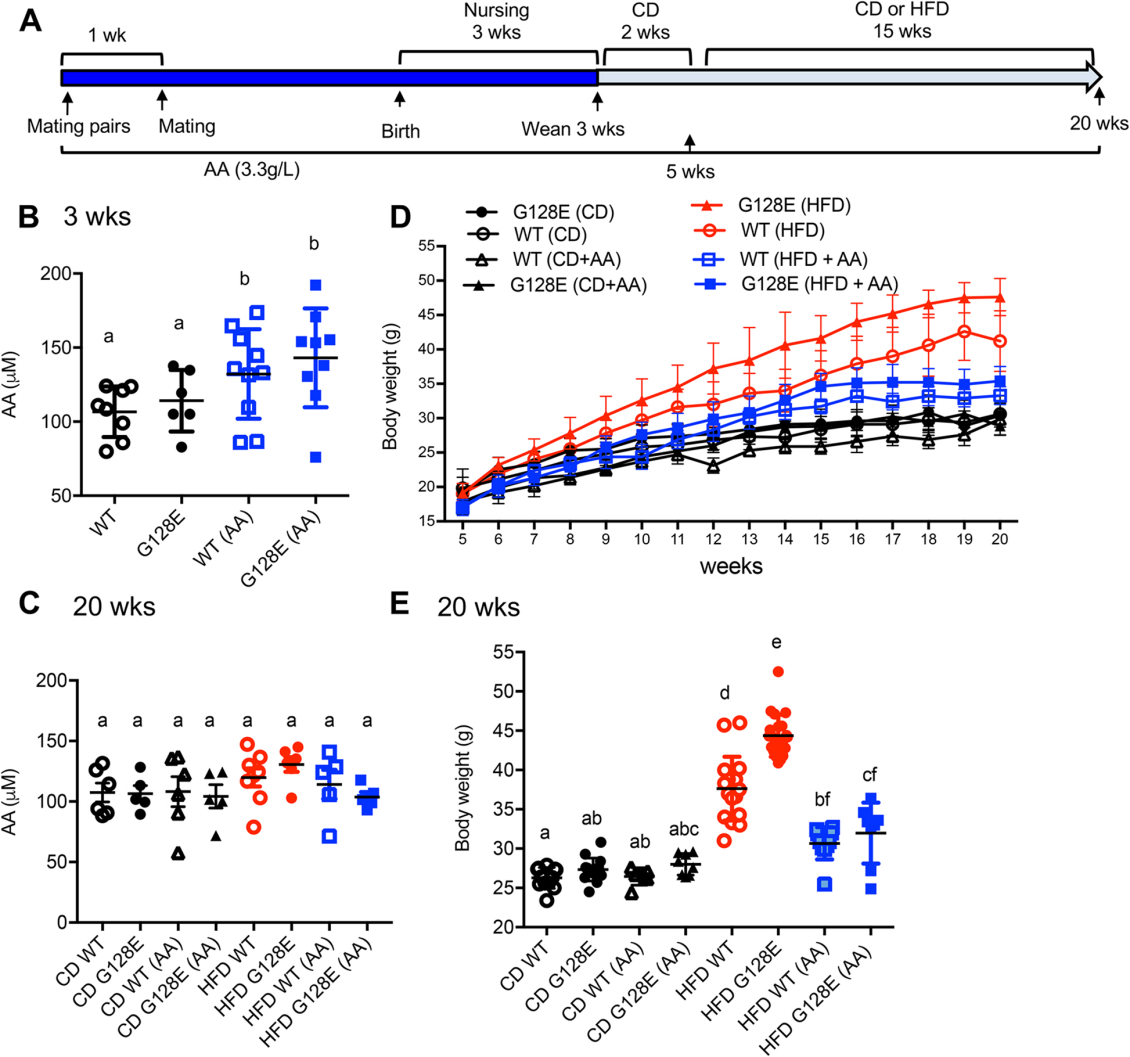
Ascorbic acid (AA) supplementation reduces HFD-induced weight gain. **A** Experimental overview. *GLUT10*^*G128E*^ or WT mating pairs, pregnant dams, nursing mothers, and weaned mice were supplied with drinking water supplemented with or without AA (3.3 g/L). The weaned mice were fed with normal diet (CD) for 2 weeks and then assigned to a CD or HFD from 5 to 20 weeks of age. **B** and **C** AA supplementation increases serum AA levels in mice at 3 weeks of age. Serum plasma AA levels were determined at 3 weeks of age (**B**) and 20 weeks of age (**C**) using the Ascorbic Acid Assay Kit. **D** and **E** AA supplementation reduces body weight gains in both WT and *GLUT10*^*G128E*^ mice. **D** Body weights were determined at indicated ages; *n* = 20 mice per group. **E** Fasting body weight at 20 weeks of age. The data are shown as mean ± SEM. Statistical significance was determined by one-way analysis of variance (ANOVA) followed by Tukey’s test for multiple comparisons. The compact letter display indicates significant differences in pairwise comparisons; groups with different letters are significantly different

To evaluate the effects of AA supplementation on metabolism in WT and *GLUT10*^*G128E*^ mice on a CD or HFD, we began supplementing the drinking water (3.3 g/L AA) of breeding pairs, nursing females, and mice after weaning. This protocol of AA supplementation was previously demonstrated to maintain optimal physiological AA levels (75 μm in serum) in AA synthesis-deficient mice [25]. Male mice were then placed on a CD or HFD at 5 weeks of age (Fig. 1A). We first analyzed the effect of AA supplementation on serum AA levels in WT and *GLUT10*^*G128E*^ mice at 3 and 20 weeks of age. We found that AA supplementation of pregnant and nursing female mice led to significantly increased serum AA levels in both WT and *GLUT10*^*G128E*^ pups at 3 weeks of age (Fig. 1B), even though mice can synthesize AA de novo [26]. In contrast, no significant differences were observed in serum AA levels among the different genotype or diet groups at 20 weeks of age, although AA supplementation was continued (Fig. 1C). Thus, AA supplementation in drinking water of breeding pairs and nursing female mice increased serum AA levels in the progeny, but AA supplementation of weaned mice did not further increase the serum AA levels in both WT and *GLUT10*^*G128E*^ mice.

We then compared the effects of AA on body weight and metabolism-related parameters in CD- or HFD-fed WT and *GLUT10*^*G128E*^ mice. *GLUT10*^*G128E*^ mice gained more weight on a HFD than did WT mice (Fig. 1 D and E) [10]. While AA effectively reduced the HFD-induced body weight gain in both HFD-fed WT mice (Fig. 1 D and E), the supplementation more readily prevented HFD-induced body weight gain in *GLUT10*^*G128E*^ mice than in WT mice (Fig. 1 D and E). AA has no effect on body weight in either WT mice or *GLUT10*^*G128E*^ mice on CD (Fig. 1 D and E). Of note, AA supplementation did not significantly affect food intake, physical activity (walking and resting times), or energy expenditure (VO2, VCO2, RER, and heat production) in mice of either genotype on a HFD (Fig. S1).

**Fig. 2:**
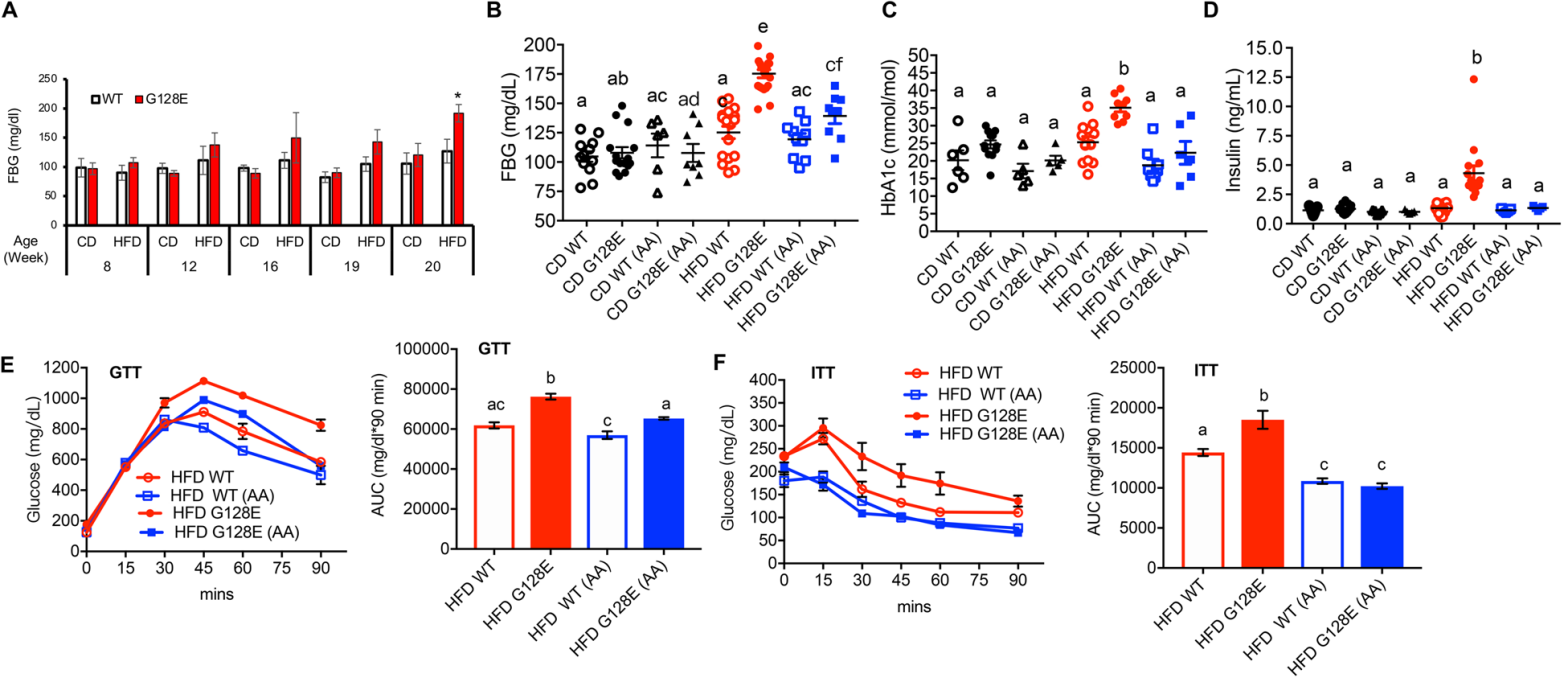
AA supplementation rescues HFD-induced metabolic dysregulation in *GLUT10*^*G128E*^ mice. Mice were treated as described in Fig. 1A. **A** HFD feeding significantly increased fasting blood glucose (FBG) levels in *GLUT10*^*G128E*^ mice at 20 weeks of age. FBG levels were determined at the indicated time point in CD- and HFD-fed WT and *GLUT10*^*G128E*^ mice. The data are shown as mean ± SEM. Statistical significance was determined by a two-tailed Student’s *t*-test. *P < 0.05. **B**–**D** AA supplementation improves metabolism-related readouts in HFD-fed *GLUT10*^*G128E*^ mice. Data were collected from mice at the conclusion of feeding (20 weeks of age). **B** Fasting glucose, **C** fasting HbA1c levels, and **D** fasting insulin levels. **E** and **F** AA supplementation had more pronounced effects on improving glucose tolerance and insulin sensitivity in HFD-fed *GLUT10*^*G128E*^ mice. **E** Glucose tolerance test (GTT) was performed on 16-week-old mice, and **F** insulin tolerance test (ITT) was performed on 18-week-old animals. Right panels in **E** and **F** show the areas under the GTT and ITT curves (AUC), respectively. The AUC were calculated using GraphPad Prism 7 software. *n* = 4 mice per group. **B–F** The data are shown as mean ± SEM. Statistical significance was determined by one-way ANOVA followed by Tukey’s test for multiple comparisons. The compact letter display indicates significant differences in pairwise comparisons; groups with different letters are significantly different

We then analyzed the effects of AA on the metabolic consequences in CD- or HFD-fed WT and *GLUT10*^*G128E*^ mice. We first monitored the changes of fasting blood glucose (FBG) levels in the mice after HFD feeding. The FBG levels were significantly higher in HFD-fed *GLUT10*^*G128E*^ mice than in HFD-fed WT mice after 15 weeks of HFD-feeding (20 weeks of age) (Fig. 2A). We therefore analyzed the effects of AA supplementation on metabolism by measuring the metabolic parameters at 20 weeks of age in CD- or HFD-fed WT and *GLUT10*^*G128E*^ mice. The readouts included FBG levels, HbA1c levels (glycated hemoglobin levels, an indicator of the daily averaged blood glucose levels [27]), and insulin levels. At this time point, FBG levels, HbA1c levels, and insulin levels were significantly increased in HFD-fed *GLUT10*^*G128E*^ mice when compared with HFD-fed WT mice (Fig. 2B, C, and D)[10], and AA supplementation attenuated HFD-induced increases in FBG, HbA1c, and insulin levels in *GLUT10*^*G128E*^ mice (Fig. 2 B, C, and D). Moreover, AA significantly improved the HFD-induced glucose intolerance and insulin resistance in *GLUT10*^*G128E*^ mice, as measured by the glucose tolerance test (GTT) and insulin resistance test (ITT), respectively (Fig. 2 E and F). Although AA had no significant effects on FBG, HbA1c, or insulin levels in HFD-fed WT mice, the supplementation did significantly improve insulin resistance in HFD-fed WT mice (Fig. 2F). Nevertheless, the improvement in HFD-fed *GLUT10*^*G128E*^ mice was more prominent than the improvement seen in HFD-fed WT (Fig. 2F). Taken together, these results suggest that AA supplementation has especially pronounced effects on attenuating the predisposition of HFD-induced metabolic dysregulation in *GLUT10*^*G128E*^ mice.

### AA supplementation reduces HFD-induced eWAT inflammation and improves adipokine dysregulation in *GLUT10*^*G128E*^ mice

**Fig. 3:**
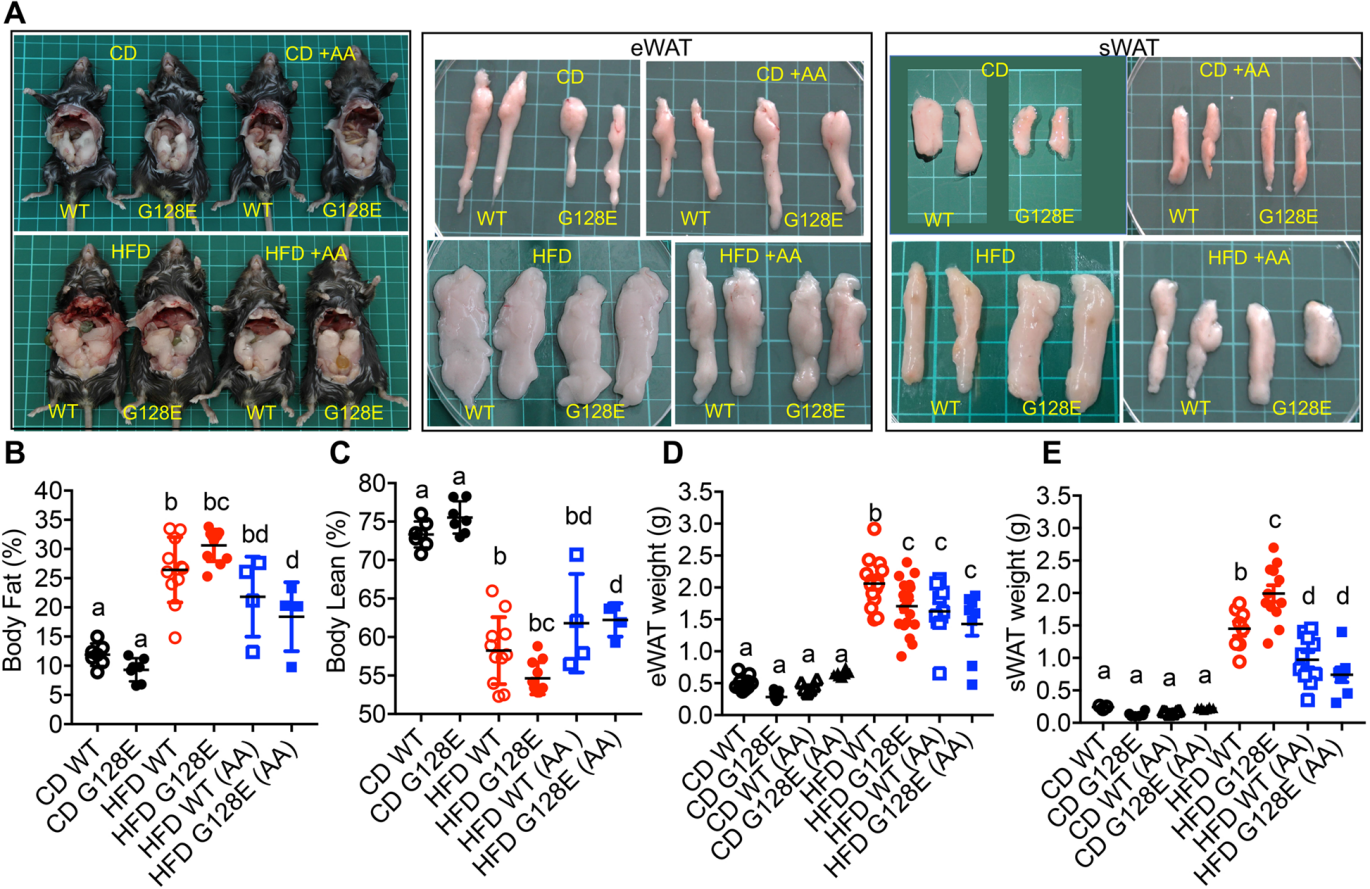
AA supplementation reduces body fat composition and WAT weights in HFD-fed mice. Mice were treated as described in Fig. 1A. Data were collected from mice at the conclusion of feeding (20 weeks of age). **A** Representative photographs of mice, eWAT and sWAT from experimental mice. Each square on the green mat is 1 cm × 1 cm. **B** Body fat and **C** body lean compositions. **D** eWAT weights and **E** sWAT weights. The data are shown as mean ± SEM. Statistical significance was determined by one-way ANOVA followed by Tukey’s test for multiple comparisons. The compact letter display indicates significant differences in pairwise comparisons; groups with different letters are significantly different

We then sought to elucidate how AA preferentially improves HFD-induced metabolic dysregulation in *GLUT10*^*G128E*^ mice. First, we determined the effects of AA supplementation on overall body fat and lean compositions in WT and *GLUT10*^*G128E*^ mice on a HFD. The AA group had a trend toward reduced body fat composition in HFD-fed WT mice, but the difference did not reach statistical significance (Fig. 3 A and B). In contrast, AA supplementation significantly reduced the body fat composition in HFD-fed *GLUT10*^*G128E*^ mice (Fig. 3 A and B). Additionally, HFD reduced the body lean composition in both WT and *GLUT10*^*G128E*^ mice, but AA only significantly attenuated the reduction in body lean composition in HFD-fed *GLUT10*^*G128E*^ mice (Fig. 3C). We therefore analyzed AA effects on two major fat pads, epididymal WAT (eWAT) and subcutaneous inguinal WAT (sWAT) in CD- or HFD-fed WT and *GLUT10*^*G128E*^ mice. AA had no effect on the weight of eWATs in CD-fed WT and *GLUT10*^*G128E*^ mice. AA supplementation reduced the weight of eWATs in HFD-fed WT mice, but not in HFD-fed *GLUT10*^*G128E*^ mice (Fig. 3D). Notably, the weight of sWAT was highly increased in HFD-fed *GLUT10*^*G128E*^ mice compared with HFD-fed WT mice, and AA supplementation has more pronounced effects in reducing the weight of sWAT in HFD-fed *GLUT10*^*G128E*^ mice than in WT mice.

**Fig. 4:**
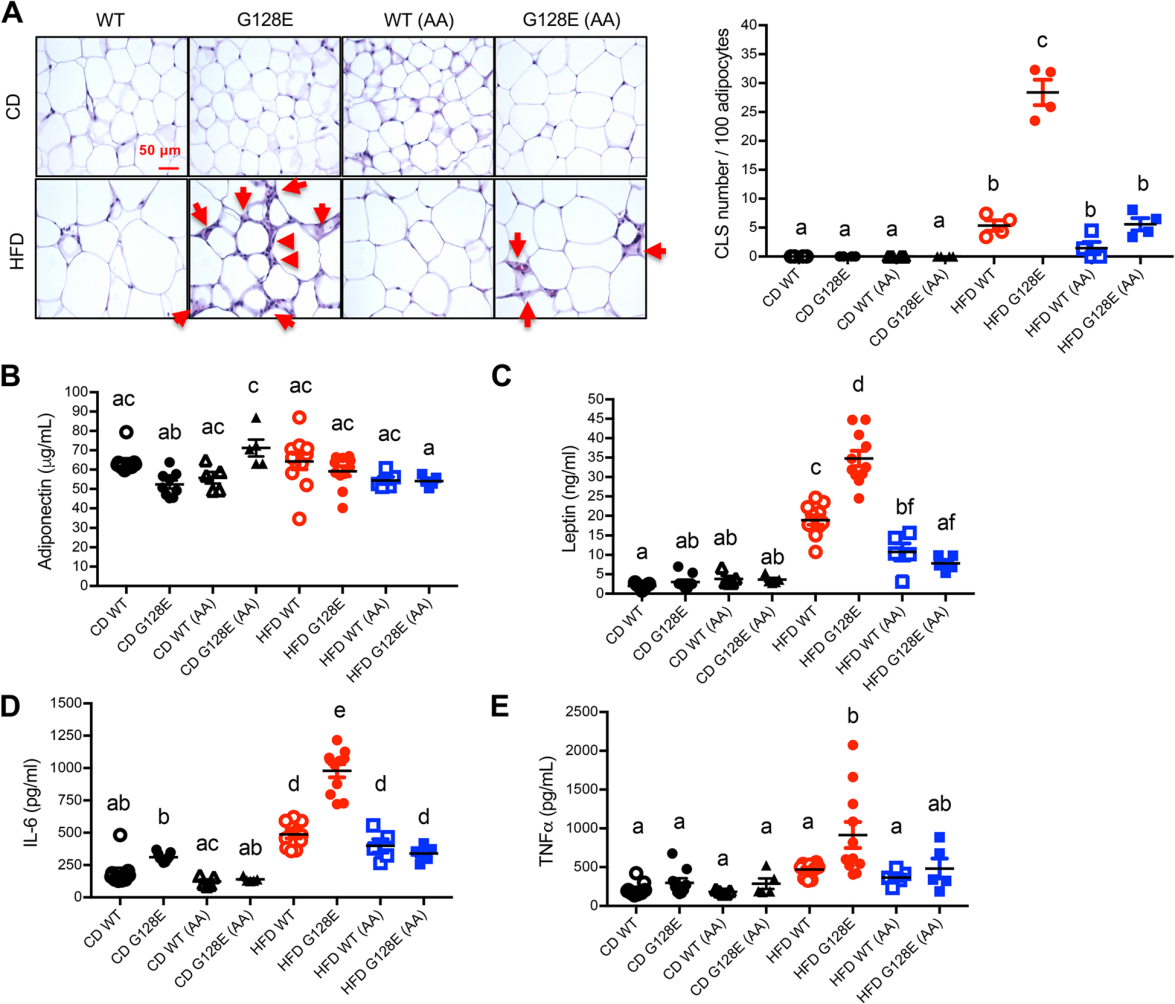
AA supplementation reduces HFD-induced eWAT inflammation and improves adipokine dysregulation in *GLUT10*^*G128E*^ mice. Mice were treated as described in Fig. 1A. Data were collected from mice at the conclusion of feeding (20 weeks of age). **A** AA supplementation reduces crown-like structures (CLSs) in eWAT of HFD-fed *GLUT10*^*G128E*^ mice. CLSs are a hallmark of eWAT inflammation. The eWAT sections were examined by H&E staining. Representing images are shown, and the red arrows point to a presumptive CLSs surrounding an adipocyte (left panel). The frequency of CLSs in eWAT sections were analyzed using ImageJ [54] and presented as CLS numbers per 100 adipocytes (right panel); *n* = 4 mice per group. **B**–**E** AA supplementation improves HFD-induced adipokine dysregulation in *GLUT10*^*G128E*^ mice. The serum levels of **B** adiponectin, **C** leptin, **D**IL-6, and **E** TNFα were determined. The data are shown as mean ± SEM. Statistical significance was determined by one-way ANOVA followed by Tukey’s test for multiple comparisons. The compact letter display indicates significant differences in pairwise comparisons; groups with different letters are significantly different

We have demonstrated that HFD feeding specifically induces inflammation and fibrosis in eWAT (a type of visceral fat) of *GLUT10*^*G128E*^ mice, but HFD does not induce a similar response in sWAT of *GLUT10*^*G128E*^ mice or the eWAT and sWAT of WT mice (Fig. S2) [10], even though the weight of sWAT was significantly increased in HFD-fed *GLUT10*^*G128E*^ mice. As central obesity (overaccumulation of visceral fat) is associated with local and systemic inflammation and predisposes individuals to metabolic dysregulation [6, 28], we therefore analyzed the AA effects on eWAT in HFD-fed WT and *GLUT10*^*G128E*^ mice. To evaluate the effects of AA supplementation on HFD-induced inflammation in eWAT, we first examined the crown-like structures (CLSs) that surround dead adipocytes and are indicative of inflammation in WAT [29]. No CLSs were observed in CD-fed mice, whereas CLSs were more frequently found in eWAT of HFD-fed *GLUT10*^*G128E*^ mice compared with HFD-fed WT mice (Fig. 4A). AA supplementation reduced the HFD-induced increases in CLSs within eWAT of *GLUT10*^*G128E*^ mice (Fig. 4A). In contrast, no CLSs were observed in sWAT of either HFD-fed WT or HFD-fed *GLUT10*^*G128E*^ mice (Fig. S2). Furthermore, AA supplementation did not affect the size or structure of adipocytes in sWAT of HFD-fed WT mice or HFD-fed *GLUT10*^*G128E*^ mice (Fig. S2). Thus, we conclude that AA supplementation has significant effects on reducing HFD-induced inflammation in *GLUT10*^*G128E*^ eWATs.

The eWAT inflammation can change the expression of adipokines and predispose individuals to metabolic dysregulation [6, 28]. We therefore determined AA effects on systemic adipokine levels in CD- and HFD-fed WT and *GLUT10*^*G128E*^ mice by analyzing the serum levels of adipokines that control systemic energy homeostasis, including adiponectin, leptin, interleukin-6 (IL-6), and tumor necrosis factor-α (TNF-α); these adipokines were previously shown to be highly dysregulated in eWAT of HFD-fed *GLUT10*^*G128E*^ mice [10]. Notably, AA supplementation only increased the serum levels of adiponectin, a protective adipokine in CD-fed *GLUT10*^*G128E*^ mice (Fig. 4B). In contrast, AA had no effect on the serum levels of adiponectin in HFD-fed *GLUT10*^*G128E*^ or in CD- and HFD-fed WT mice (Fig. 4B). AA supplementation significantly suppressed the elevated serum leptin levels, a cytokine correlated with body fat composition, and this suppression was to a higher extent in HFD-fed *GLUT10*^*G128E*^ mice compared with HFD-fed WT mice (Fig. 4C). Most importantly, AA supplementation significantly suppressed the serum levels of inflammatory cytokine, IL-6, which were highly elevated in HFD-fed *GLUT10*^*G128E*^ mice (Fig. 4D). Also, AA supplementation caused a nonsignificant trend toward suppression of serum TNF-α levels (the other inflammatory cytokine) elevated in HFD-fed *GLUT10*^*G128E*^ mice (Fig. 4E). In contrast, AA supplementation did not affect the serum levels of IL-6 and TNF-α in CD- or HFD-fed WT mice (Fig. 4 D and E).

Altogether, these findings lead us to conclude that AA supplementation counteracts the predisposition of *GLUT10*^*G128E*^ mice to HFD-induced eWAT inflammation and adipokine dysregulation.

### AA supplementation reduces HFD-induced ectopic lipid accumulation in *GLUT10*^*G128E*^ mice

**Fig. 5:**
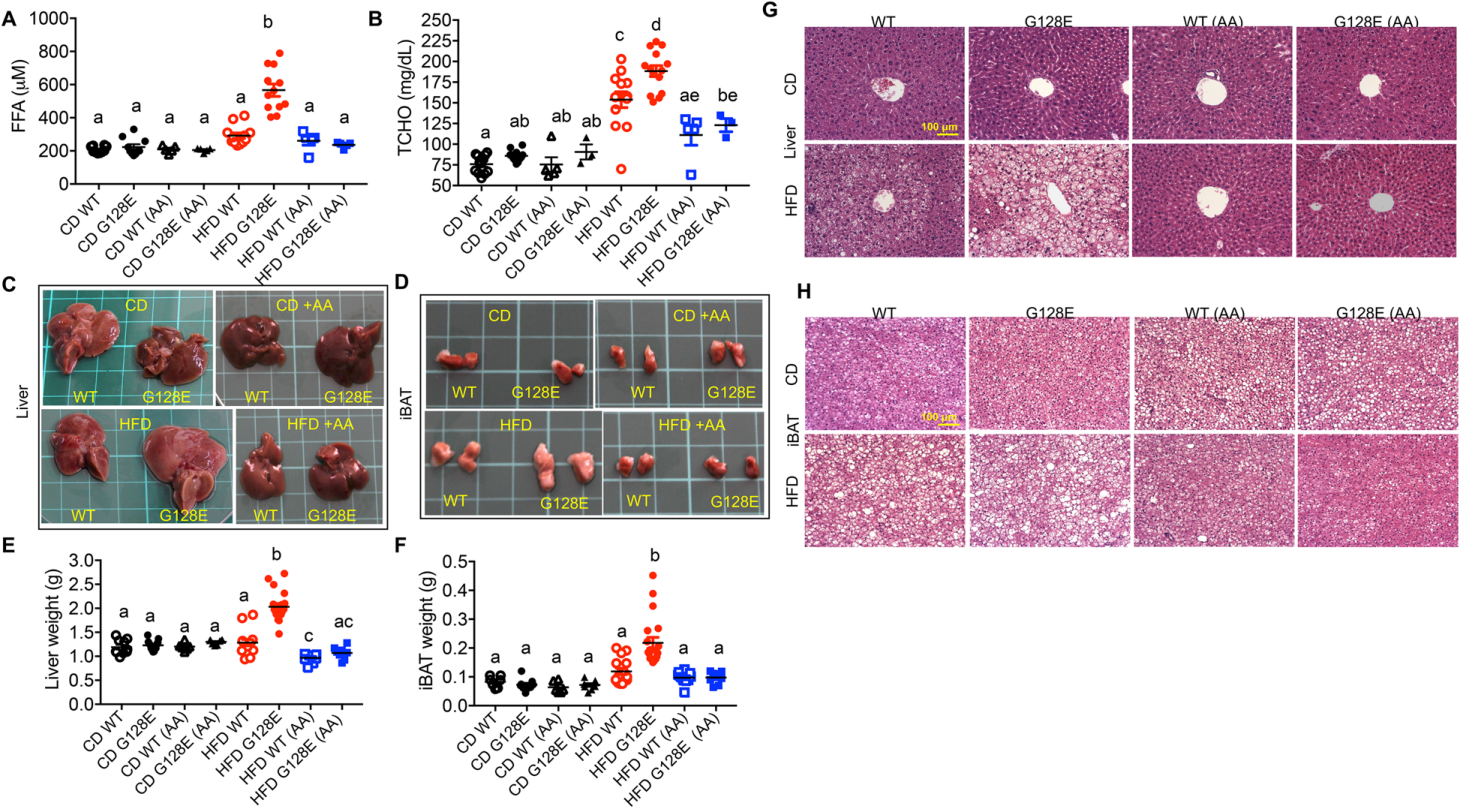
AA supplementation reduces HFD-induced ectopic lipid accumulation in *GLUT10*^*G128E*^ mice. Mice were treated as described in Fig. 1A. Data were collected from mice at the conclusion of feeding (20 weeks of age). **A**–**B** AA supplementation reduces HFD-induced free fatty acid (FFA) levels and total cholesterol (TCHO) levels in *GLUT10*^*G128E*^ mice. **A** FFA levels and **B** TCHO levels in serum were determined. **C**–**H** AA supplementation rescues HFD-induced ectopic lipid accumulation in liver and iBAT. Representative photographs of **C** livers and **D** iBAT from experimental mice. Each square on the green mat is 1 cm × 1 cm. **E** Liver weights. **F** iBAT weights. H&E staining of **G** liver sections and **H** iBAT sections. The data are shown as mean ± SEM. Statistical significance was determined by one-way ANOVA followed by Tukey’s test for multiple comparisons. The compact letter display indicates significant differences in pairwise comparisons; groups with different letters are significantly different

eWAT inflammation and adipokine dysregulation can contribute to increased serum levels of free fatty acids (FFA) and total cholesterol (TCHO), leading to lipid deposition in other organs, including liver and interscapular brown adipose tissue (iBAT) [30–32]. We have demonstrated that HFD-fed *GLUT10*^*G128E*^ mice have increased serum levels of FFA and TCHO and increased lipid accumulation in the liver and iBAT, as demonstrated by increased tissue size and weight, and more frequent appearance of fat vacuoles in tissue sections [10]. Thus, we set out to determine the effects of AA supplementation on HFD-induced ectopic lipid accumulation in *GLUT10*^*G128E*^ mice by examining these parameters. Notably, AA supplementation reduced the HFD-induced serum levels of FFA and TCHO in *GLUT10*^*G128E*^ mice (Fig. 5 A and B). Furthermore, AA supplementation reduced the sizes and tissue weights of the liver and iBAT and appearance of fat vacuoles in these tissues from HFD-fed *GLUT10*^*G128E*^ mice (Fig. 5 C–H). In contrast, AA had no significant effects on serum FFA levels or sizes and tissue weights of the liver and iBAT and appearance of fat vacuoles in these tissues in HFD-fed WT mice (Fig. 5 A–H). Thus, we conclude that AA supplementation prevented the HFD-induced ectopic lipid accumulation in *GLUT10*^*G128E*^ mice.

### AA supplementation improves eWAT development in *GLUT10*^*G128E*^ mice

**Fig. 6:**
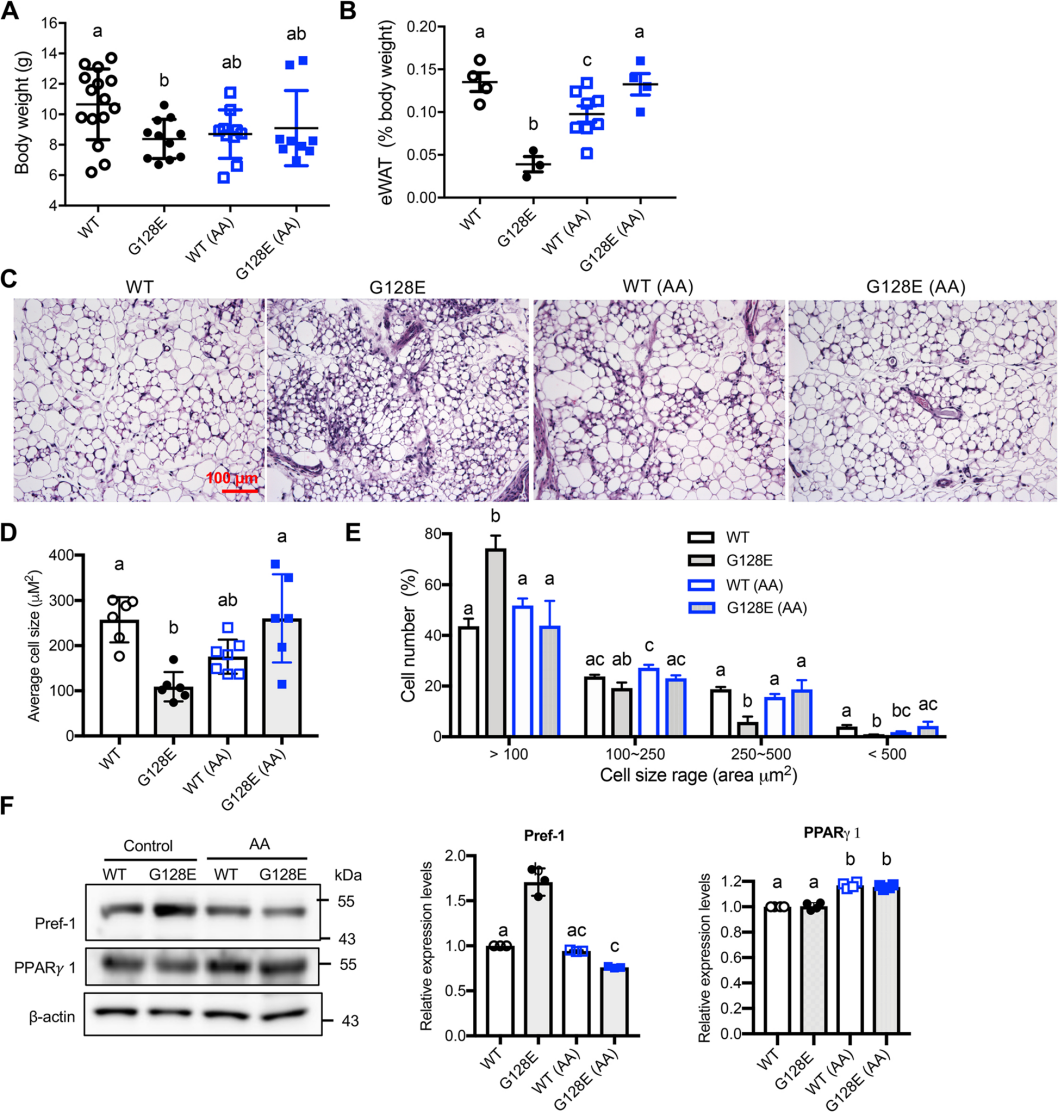
AA supplementation improves eWAT development in *GLUT10*^*G128E*^ mice. Mice were treated as described in Fig. 1A. Data were collected from pups at 3 weeks of age. **A** and **B** AA supplementation increases percentage of eWAT weight of *GLUT10*^*G128E*^ mice. **A** Body weight and **B** eWAT percent of total body weight were measured. **C**–**E** AA supplementation increases adipocytes size in eWAT of *GLUT10*^*G128E*^ mice. **C** Representative photographs of hematoxylin and eosin (H&E) staining of eWAT sections. The cross-sectional area of adipose cells in eWAT is presented as **D** average size of cells and **E** the percentage of cells in the given size range. In **D** and **E**, *n* = 6 mice per group; more than 1000 adipocytes were analyzed in each mouse. **F** AA supplementation reduces Pref-1 protein levels in eWAT of *GLUT10*^*G128E*^ mice. The protein levels of Pref-1 and PPARγ 1 in eWAT were analyzed by Western blotting. Protein samples from 6 mice per group were pooled. The protein levels were quantified, normalized to β-actin levels, and compared to WT no-AA-treated controls. The data are shown as mean ± SEM from triple repeats of Western blotting experiments of the pooled samples. Statistical significance was determined by one-way ANOVA followed by Tukey’s test for multiple comparisons. The compact letter display indicates significant differences in pairwise comparisons; groups with different letters are significantly different

Next, we sought to determine how AA reduces HFD-induced eWAT inflammation and improves subsequent metabolic dysregulation in *GLUT10*^*G128E*^ mice. As *GLUT10*^*G128E*^ mice have compromised eWAT development, which plays a critical role in predisposing the mice to HFD-induced metabolic dysregulation [10], we decided to test whether AA supplementation could improve compromised eWAT development in *GLUT10*^*G128E*^ mice. The eWAT deposits arise during late-embryonic and neonatal development [33]. We found AA-mediated improvements in early eWAT development by monitoring the weight and histology of eWAT at 3 weeks of age. AA supplementation in pregnant and nursing female mice did not affect body weights of either WT or *GLUT10*^*G128E*^ pups (Fig. 6A). Notably, AA supplementation reversed the decreased weight of eWAT in *GLUT10*^*G128E*^ pups (Fig. 6B). Furthermore, AA supplementation also reversed the reduction of average adipocyte size in *GLUT10*^*G128E*^ eWAT, by reducing the percentage of small adipocytes (< 100 area μm^2^) and increasing the percentage of large adipocytes (> 250 area μm^2^), according to quantification of adipocyte size in the eWAT sections (Fig. 6 C–E). In contrast, AA supplementation did not affect the weight of eWAT, nor did it affect the average size or size range of adipocytes in eWAT of WT mice (Fig. 6 B–E). Thus, AA supplementation reversed the reduced weight and reduced adipocyte size in eWAT of *GLUT10*^*G128E*^ pups.

We next examined whether increased adipogenesis is also involved in AA-mediated reversal of the reduced weight of eWATs in *GLUT10*^*G128E*^ pups. In previous work, AA supplementation in cultured cells was found to differentially induces adipogenesis in GLUT10-deficient and control preadipocytes [10]. In particular, AA supplementation was found to induce more pronounced effects on adipogenesis in GLUT10-deficient preadipocytes compared with controls, including mouse embryonic fibroblasts (MEFs) from *GLUT10*^*G128E*^ mice as well as GLUT10-knockdown preadipocytes (3T3-L1 cells) [10]. We then determined the AA effects on adipogenesis in vivo by examining the expression of a preadipocyte marker, preadipocyte factor 1 (Pref-1), and a key adipogenic transcription factor, peroxisome proliferator-activated receptor gamma 1 (PPARγ 1), in eWATs of WT and *GLUT10*^*G128E*^ mice at 3 weeks of age. Pref-1 is highly expressed in preadipocytes and absent after adipocyte differentiation [34]. *GLUT10*^*G128E*^ eWATs had higher levels of Pref-1 protein than did WT eWATs, and AA supplementation reduced the Pref-1 protein levels in *GLUT10*^*G128E*^ eWATs (Fig. 6F). These results suggest that more preadipocytes existed in *GLUT10*^*G128E*^ eWATs than WT eWATs, and AA supplementation reduced the preadipocytes in *GLUT10*^*G128E*^ eWATs. Furthermore, AA supplementation increased the expression levels of PPARγ 1 in eWATs of GLUT10^G128E^ mice and WT mice. These in vivo findings together with previous in vitro findings lead us to conclude that AA supplementation has more beneficially effects, in terms of promoting adipogenesis and reducing undifferentiated preadipocytes in eWATs of *GLUT10*^*G128E*^ than in eWATs of WT mice.

## Discussion

**Fig. 7:**
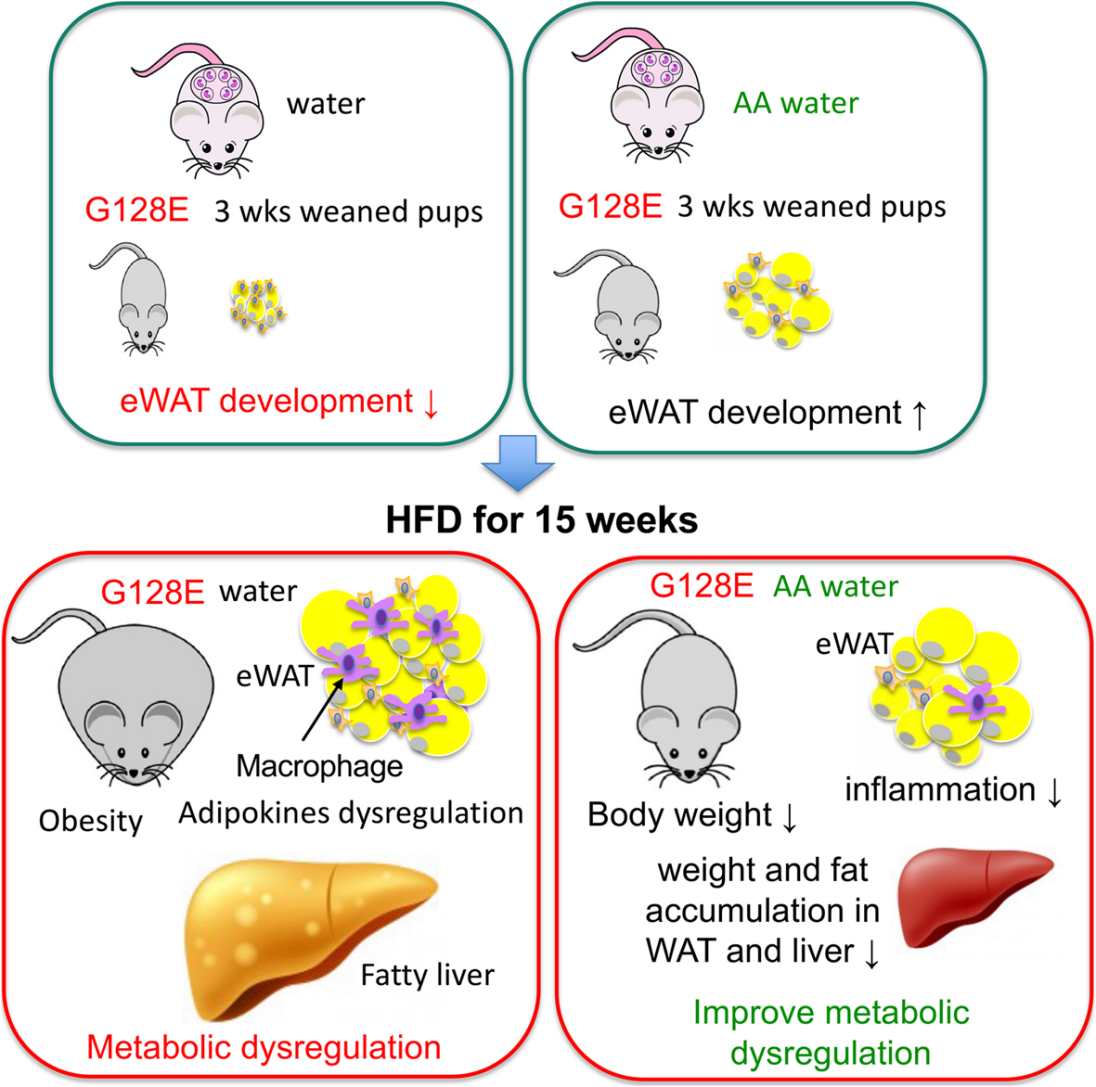
Model of AA-mediated attenuation of HFD-induced metabolic dysregulation in *GLUT10*^*G128E*^ mice. AA supplementation in pregnant dams, nursing mothers, and weaned mice rescues eWAT development in *GLUT10*^*G128E*^ pups and attenuates later HFD-induced eWAT inflammation and metabolic dysregulation in *GLUT10*^*G128E*^ mice

The development of T2DM involves interactions between genetic and environmental factors, and transitions in customary dietary patterns (e.g., switch to HFD) have greatly contributed to the increased prevalence of obesity and accelerated the spread of T2DM epidemic worldwide. At least partly based on its antioxidant properties, AA has been considered as a complementary nutritional treatment for T2DM. However, evidence for the significance and beneficial effect of AA in T2DM has thus far been inconclusive [8, 9]. We suspect that one reason for this inconclusive evidence is that combined genetic and dietary factors may greatly influence AA effects on metabolism. To test this idea, we evaluated the effects of AA supplementation on metabolism in the context of combined genetic and dietary risk factors, i.e., mice carrying an orthologous human *GLUT10*^*G128E*^ variant and feeding with HFD. We chose this combination because genetic polymorphisms in GLUT10 gene are associated with a T2DM intermediate phenotype in nondiabetic population, and *GLUT10*^*G128E*^ mice are highly sensitive to HFD-induced metabolic dysregulation [10]. Our experiments demonstrated that AA is more beneficial in *GLUT10*^*G128E*^ mice than in WT mice, in terms of attenuating HFD-induced obesity and metabolic dysregulation. The mechanism of protection is partly through AA-mediated improvements to compromised eWAT development in *GLUT10*^*G128E*^ pups, which diminishes later HFD-induced eWAT inflammation and metabolic dysregulation in *GLUT10*^*G128E*^ mice (Fig. 7). Together, our findings support the idea that the individual gene variants and dietary patterns should be taken into account when considering AA for T2DM prevention and treatment. Our study also suggests that proper WAT development at a young age is essential for metabolic regulation later in life. Moreover, higher systemic AA levels might facilitate proper WAT development and protect against HFD-induced metabolic dysregulation in individuals with other risk factors, as many genetic variants and environmental factors can compromise AA status [35, 36]. It is possible that higher levels of AA intake may be beneficial to maintain metabolic homeostasis in these populations.

We found that AA supplementation significantly increases serum AA levels in pups at 3 weeks of age (before weaning). However, we did not observe significantly increased serum AA levels in mice at 20 weeks of age, even though the AA supplementation was continued (Fig. 1). Like the majority of mammals, mice can synthesize AA in the liver [24]. The animals begin to synthesize AA in fetal stages, around the middle gestation. However, the AA synthesis ability in fetal and young animals (before weaning) remains comparatively low, with a major increase in AA synthesis occurring at about the time of weaning [37, 38, 39]. As AA is critical to the rapid growth and development in late gestation fetus and early postnatal periods, high levels of maternal AA are transferred to the fetus and to young pups via milk in order to satisfy the AA demands [39]. Based on this timeline, we expect that the increases of serum AA levels we observed in mice at 3 weeks of age may have been largely due to contributions from the dam. Adult mice have relatively high capacities to synthesis AA [40], and the animals might also have homeostatic mechanisms that mask the effects of AA supplementation at adult stages. Nevertheless, the detailed mechanisms of AA homeostasis in adult mice remain unclear. Interestingly, AA supplementation in nursing females has been shown to have beneficial effects on the development of young animals, despite the endogenous synthesis of AA in dam and pups [39]. Taking these previous studies together with our findings, AA supplementation in pregnant and nursing females appears to support rapid fetal and postnatal growth, when high levels of AA are required.

We found that AA supplementation differentially affects HFD-induced obesity and metabolic dysregulation in WT and *GLUT10*^*G128E*^ mice. The mechanisms by which AA reduces obesity and improves metabolic dysregulation in different obese models may be complex. AA supplementation in pregnant and nursing females significantly increases serum AA levels and improves the development of eWATs in the *GLUT10*^*G128E*^ pups (Figs. 1 and 6). WAT actively regulates whole-body energy homeostasis by storing lipids and secreting adipokines [6], and congenital lipodystrophy (impaired WAT development) can lead to almost all features of metabolic syndrome [41, 42]. Therefore, AA-mediated improvements to compromised WAT development in *GLUT10*^*G128E*^ pups might be largely responsible for the later attenuation of HFD-induced metabolic dysregulation in *GLUT10*^*G128E*^ mice. As such, these results highlight the importance of proper WAT development on lifelong metabolic regulation.

Several mechanisms have been proposed to link AA with reduced obesity, and metabolic dysregulation has been proposed in different rodent models of obesity. AA supplementation has been shown to combat obesity and metabolic syndrome in cafeteria diet-induced obese rats [43], obese mice (*ob/ob* mice) [44], and ovariectomized rats [45], without affecting food intake. It has been postulated that AA effects might be in part through anti-oxidative and anti-inflammatory properties [46–49]. Furthermore, evidence from cultured cells suggests that AA is involved in modulating intracellular fat accumulation [45, 50]. Since no obvious signs of inflammation were observed in our HFD-fed WT mice, AA reductions in lipid accumulation might be a key reason for its ability to reduce body weight and body fat in the HFD-fed WT mice. In addition to improving compromised WAT development in *GLUT10*^*G128E*^ pups, the anti-oxidative and anti-inflammatory activities may participate with AA-mediated reductions in lipid accumulation to generate the final outcome of reduced obesity and improved metabolic dysregulation in HFD-fed *GLUT10*^*G128E*^ mice.

AA is also an enzyme cofactor that is required for many important biological functions [51]. In this capacity, AA regulates DNA demethylation, histone demethylation, and synthesis of collagen and carnitine [51, 52]. We have demonstrated that AA supplementation induces adipogenesis through increasing DNA demethylation in the regulatory regions of two central adipogenesis-regulating transcription factors, *Cebpa* and *Pparg*, increasing their expression [10]. These results suggest that AA supplementation may promote adipogenesis to improve the development of *GLUT10*^*G128E*^ eWATs via DNA demethylation. Whether the other functions of AA might also influence adipose function and metabolism in the context of HFD feeding remains to be investigated.

In this work, we utilize *GLUT10*^*G128E*^ mice carrying an orthologous human *GLUT10*^*G128E*^ variant with compromised GLUT10 function. These mice have increased serum HbA1c levels under CD diet and are genetically predisposed to HFD-induced metabolic dysregulation. Similarly, genetic polymorphisms in the GLUT10-encoding human gene (*SLC2A10*) locus are associated with increased serum HbA1c levels in a nondiabetic population [10]. Furthermore, adipogenesis is not only reduced in *GLUT10*^*G128E*^ MEFs but also in GLUT10-knockdown 3T3-L1 cells [10]. In light of the fact that there are more than 700 identified rare variants in *SLC2A10* gene that are predicted to affect GLUT10 function, our results suggest that other variants in *SLC2A10* associated with compromised GLUT10 expression or function might also negatively affect eWAT development, and AA could potentially attenuate HFD-induced metabolic dysregulation in carriers of these variants.

## Conclusions

Overall, our findings in this study provide a proof of concept that AA has differential effects on metabolism in the presence or absence of genetic and environmental T2DM-risk factors. We expect these results will stimulate more sophisticated population studies to accurately assess whether AA is an appropriate treatment or preventative agent for T2DM in susceptible populations.

## Methods

### Mice

All animal protocols were approved by the Institutional Animal Care and Utilization Committee at Academia Sinica (Protocol no. 14–12-795). *GLUT10*^*G128E*^ mice were generated on a C3HeB/FeJ background [53] and were backcrossed to C57BL/6 J background as previously described [13]. WT and *GLUT10*^*G128E*^ mice in this study were on the C57BL/6 J background and maintained by heterozygous mattings. Mice were housed in a specific pathogen-free controlled environment with a 14-h light/10-h dark cycle at 21–23 °C. AA-supplemented groups received AA (3.3 g/L) and 0.01 mM EDTA in the drinking water, which was changed once per week. The stability of AA in drinking water was examined by determining the AA levels in the water by HPLC as described previously [15]. The level was measured daily for 7 days after the water was prepared. The AA levels in water were not changed during the monitoring period, suggesting that AA is stable under the conditions. AA supplementation was provided to breeding pairs, pregnant dams, nursing mothers, and after weaning. For diet treatments, after weaning, mice were fed with a CD for 2 weeks and then placed on a CD or HFD from 5 to 15 weeks of age (Fig. 1A). The standard rodent diet (CD) contained 13% energy from fat (LabDiet 5010 rodent Diet, PMI Nutrition International Inc., Brentwood, MO, USA), and the HFD contained 60% energy from fat (58Y1, Young Li Trading Co., New Taipei City, Taiwan). Male mice were used in this study. No data were excluded in the analyses.

### Glucose and insulin tolerance tests (GTT and ITT)

For fasting blood glucose measurements, blood samples were collected from the tail vein after overnight fasting. The GTT and ITT were performed as previously described [10]. Briefly, GTT was analyzed in mice at 16 weeks of age, and mice were fasted for 18 h before receiving an intraperitoneal injection of glucose (2 g/kg). ITT was performed on mice at 18 weeks of age; mice were fasted for 8 h, followed by an intraperitoneal injection of insulin (0.75 U/kg, Humulin R U100, Lilly, Eli and Company, Indianapolis, IN, USA). Blood samples were collected from the tail vein prior to injection and again at 15, 30, 45, 60, 75, and 90 min post-injection. Blood glucose levels were assessed using a glucometer (Accu-Chek Performa, Roche Medical Diagnostic Equipment Co., Taiwan).

### Measurement of food intake and metabolic rate

Mice were housed individually for measurement of food intake and water intake, using Tecniplast® Metabolic Cage (Tecniplast, Via I Maggio, Italy). The metabolic rate was measured using the CLAMS-home cage (CLAMS-HC) system (Columbus Instruments, Columbus, OH, USA) in the Taiwan Mouse Clinic at Academia Sinica. The first readings were taken after a 48-h acclimation period. Heat production, RER, oxygen consumption rate (VO2), and carbon dioxide production (VCO2) rates were determined. VO2, VCO2, and heat were measured every 17 min during a 76-h period at the indicated temperature and were normalized to body weight.

### Blood chemistry and adipokine assays

For blood chemistry and adipokine assays, blood was collected from cardiac puncture at the conclusion of experiments. The TCHO levels were analyzed from serum samples using Fuji biochemical slides and a Fuji Dri-Chem 4000i analyzer (Fujifilm Cooperation, Taipei, Taiwan) in the Taiwan Mouse Clinic at Academia Sinica. Plasma levels of adiponectin, leptin, IL-6, and insulin were measured using mouse ELISA kits (Merck Millipore, Taipei, Taiwan). The plasma free fatty acids were measured using an ELISA kit (ab65341, Abcam, Cambridge, MA, USA), and blood HbA1c was measured using the mouse Hemoglobin A1c (HbA1c) Assay Kit (Crystal Chem, Elk Grove Village, IL, USA).

### Serum AA measurements

Serum AA levels were determined using an Ascorbic Acid Assay Kit (Abcam, Cambridge, England, UK).

### Histological analysis and immunohistochemistry

Tissue sections were stained with hematoxylin and eosin (H&E).

### Western blot

Total protein lysates from tissues were used for analysis. Proteins were transferred to PVDF membranes (Millipore, Billerica, MA). Membranes were then incubated with the primary antibodies against Pref-1 (DLK1) (Proteintech, Rosemont, IL), PPARγ 1 (Santa Cruz, Dallas, TX), or β-actin (GeneTex, Irvine, CA) and the appropriate secondary antibodies. The signal was detected by enhanced chemiluminescence (Millipore Merck, Taipei, Taiwan).

### Body composition

Mouse body composition was analyzed with Bruker’s Minispec LF50 Body Composition Analyzer in the Taiwan Mouse Clinic at Academia Sinica.

### Statistics

Statistical analyses were performed in GraphPad Prism 7 (GraphPad Software, La Jolla, CA, USA). Data are presented as mean ± standard error of the mean (SEM). Statistical significance was determined by one-way analysis of variance (ANOVA) followed by Tukey’s test for multiple comparisons. A compact letter display was used to indicate significant differences in pairwise comparisons. *P*-values less than 0.05 were considered statistically significant.

## Supplementary Information

**Additional file 1: Figure S1.** Mice from different genotypes or treatment groups exhibit no significant differences in food intake, water intake, physical activity, or energy expenditure. **Figure S2.** AA supplementation has no obvious effect on histology of sWAT of HFD-fed *GLUT10*^*G128E*^ and WT mice.

## Abbreviations

T2DM: Type 2 diabetes mellitus
AA: Ascorbic acid, vitamin C
ATS: Arterial tortuosity syndrome
GLUT10: Glucose transporter 10
CD: Control diet
HFD: High-fat diet
WT: Wild type
WAT: White adipose tissue
eWAT: Epididymal white adipose tissue
sWAT: Subcutaneous inguinal WAT
iBAT: Interscapular brown adipose tissue
FBG: Fasting blood glucose
FFA: Free fatty acids
TCHO: Total cholesterol

## References

[1] Hu FB (2011). Globalization of diabetes: the role of diet, lifestyle, and genes. Diabetes Care.

[2] Flannick J, Florez JC (2016). Type 2 diabetes: genetic data sharing to advance complex disease research. Nat Rev Genet.

[3] DeFronzo RA, Ferrannini E, Groop L, Henry RR, Herman WH, Holst JJ, Hu FB, Kahn CR, Raz I, Shulman GI (2015). Type 2 diabetes mellitus. Nat Rev Dis Primers.

[4] McAllister K, Mechanic LE, Amos C, Aschard H, Blair IA, Chatterjee N, Conti D, Gauderman WJ, Hsu L, Hutter CM (2017). Current challenges and new opportunities for gene-environment interaction studies of complex diseases. Am J Epidemiol.

[5] Pollack RM, Donath MY, LeRoith D, Leibowitz G (2016). Anti-inflammatory agents in the treatment of diabetes and its vascular complications. Diabetes Care.

[6] Choe SS, Huh JY, Hwang IJ, Kim JI, Kim JB (2016). Adipose tissue remodeling: its role in energy metabolism and metabolic disorders. Front Endocrinol (Lausanne).

[7] Kloting N, Bluher M (2014). Adipocyte dysfunction, inflammation and metabolic syndrome. Rev Endocr Metab Disord.

[8] Tareke AA, Hadgu AA (2021). The effect of vitamin C supplementation on lipid profile of type 2 diabetic patients: a systematic review and meta-analysis of clinical trials. Diabetol Metab Syndr.

[9] Wong SK, Chin KY, Ima-Nirwana S (2020). Vitamin C: A review on its role in the management of metabolic syndrome. Int J Med Sci.

[10] Jiang CL, Jen WP, Tsao CY, Chang LC, Chen CH, Lee YC (2020). Glucose transporter 10 modulates adipogenesis via an ascorbic acid-mediated pathway to protect mice against diet-induced metabolic dysregulation. PLoS Genet.

[11] Coucke PJ, Willaert A, Wessels MW, Callewaert B, Zoppi N, De Backer J, Fox JE, Mancini GM, Kambouris M, Gardella R (2006). Mutations in the facilitative glucose transporter GLUT10 alter angiogenesis and cause arterial tortuosity syndrome. Nat Genet.

[12] Nemeth CE, Nemoda Z, Low P, Szabo P, Horvath EZ, Willaert A, Boel A, Callewaert BL, Coucke PJ, Colombi M (2019). Decreased nuclear ascorbate accumulation accompanied with altered genomic methylation pattern in fibroblasts from arterial tortuosity syndrome patients. Oxid Med Cell Longev.

[13] Syu YW, Lai HW, Jiang CL, Tsai HY, Lin CC, Lee YC (2018). GLUT10 maintains the integrity of major arteries through regulation of redox homeostasis and mitochondrial function. Hum Mol Genet.

[14] Nemeth CE, Marcolongo P, Gamberucci A, Fulceri R, Benedetti A, Zoppi N, Ritelli M, Chiarelli N, Colombi M, Willaert A (2016). Glucose transporter type 10 - lacking in arterial tortuosity syndrome - facilitates dehydroascorbic acid transport. FEBS Lett.

[15] Lee YC, Huang HY, Chang CJ, Cheng CH, Chen YT (2010). Mitochondrial GLUT10 facilitates dehydroascorbic acid import and protects cells against oxidative stress: mechanistic insight into arterial tortuosity syndrome. Hum Mol Genet.

[16] Ghosh S, Watanabe RM, Hauser ER, Valle T, Magnuson VL, Erdos MR, Langefeld CD, Balow J, Ally DS, Kohtamaki K (1999). Type 2 diabetes: evidence for linkage on chromosome 20 in 716 Finnish affected sib pairs. Proc Natl Acad Sci U S A.

[17] Zouali H, Hani EH, Philippi A, Vionnet N, Beckmann JS, Demenais F, Froguel P (1997). A susceptibility locus for early-onset non-insulin dependent (type 2) diabetes mellitus maps to chromosome 20q, proximal to the phosphoenolpyruvate carboxykinase gene. Hum Mol Genet.

[18] Andersen G, Rose CS, Hamid YH, Drivsholm T, Borch-Johnsen K, Hansen T, Pedersen O (2003). Genetic variation of the GLUT10 glucose transporter (SLC2A10) and relationships to type 2 diabetes and intermediary traits. Diabetes.

[19] Bento JL, Bowden DW, Mychaleckyj JC, Hirakawa S, Rich SS, Freedman BI, Segade F (2005). Genetic analysis of the GLUT10 glucose transporter (SLC2A10) polymorphisms in Caucasian American type 2 diabetes. BMC Med Genet.

[20] Mohlke KL, Skol AD, Scott LJ, Valle TT, Bergman RN, Tuomilehto J, Boehnke M, Collins FS (2005). Evaluation of SLC2A10 (GLUT10) as a candidate gene for type 2 diabetes and related traits in Finns. Mol Genet Metab.

[21] Rose CS, Andersen G, Hamid YH, Glumer C, Drivsholm T, Borch-Johnsen K, Jorgensen T, Pedersen O, Hansen T (2005). Studies of relationships between the GLUT10 Ala206Thr polymorphism and impaired insulin secretion. Diabet Med.

[22] Lin WH, Chuang LM, Chen CH, Yeh JI, Hsieh PS, Cheng CH, Chen YT (2006). Association study of genetic polymorphisms of SLC2A10 gene and type 2 diabetes in the Taiwanese population. Diabetologia.

[23] Jiang YD, Chang YC, Chiu YF, Chang TJ, Li HY, Lin WH, Yuan HY, Chen YT, Chuang LM (2010). SLC2A10 genetic polymorphism predicts development of peripheral arterial disease in patients with type 2 diabetes. SLC2A10 and PAD in type 2 diabetes. BMC Med Genet.

[24] Drouin G, Godin JR, Page B (2011). The genetics of vitamin C loss in vertebrates. Curr Genomics.

[25] Kim H, Bae S, Yu Y, Kim Y, Kim HR, Hwang YI, Kang JS, Lee WJ (2012). The analysis of vitamin C concentration in organs of gulo(-/-) mice upon vitamin C withdrawal. Immune Netw.

[26] Linster CL, Van Schaftingen E (2007). Vitamin C biosynthesis, recycling and degradation in mammals. FEBS J.

[27] Han BG, Hao CM, Tchekneva EE, Wang YY, Lee CA, Ebrahim B, Harris RC, Kern TS, Wasserman DH, Breyer MD (2008). Markers of glycemic control in the mouse: comparisons of 6-h- and overnight-fasted blood glucoses to Hb A1c. Am J Physiol Endocrinol Metab.

[28] Chait A, den Hartigh LJ (2020). Adipose tissue distribution, inflammation and its metabolic consequences, including diabetes and cardiovascular disease. Front Cardiovasc Med.

[29] Altintas MM, Azad A, Nayer B, Contreras G, Zaias J, Faul C, Reiser J, Nayer A (2011). Mast cells, macrophages, and crown-like structures distinguish subcutaneous from visceral fat in mice. J Lipid Res.

[30] Sun K, Kusminski CM, Scherer PE (2011). Adipose tissue remodeling and obesity. J Clin Invest.

[31] Olefsky JM, Glass CK (2010). Macrophages, inflammation, and insulin resistance. Annu Rev Physiol.

[32] Suganami T, Tanaka M, Ogawa Y (2012). Adipose tissue inflammation and ectopic lipid accumulation. Endocr J.

[33] Hudak CS, Gulyaeva O, Wang Y, Park SM, Lee L, Kang C, Sul HS (2014). Pref-1 marks very early mesenchymal precursors required for adipose tissue development and expansion. Cell Rep.

[34] Wang Y, Kim KA, Kim JH, Sul HS (2006). Pref-1, a preadipocyte secreted factor that inhibits adipogenesis. J Nutr.

[35] Michels AJ, Hagen TM, Frei B (2013). Human genetic variation influences vitamin C homeostasis by altering vitamin C transport and antioxidant enzyme function. Annu Rev Nutr.

[36] Carr AC, Rowe S (2020). Factors affecting vitamin C status and prevalence of deficiency: a global health perspective. Nutrients.

[37] Kratzing CC, Kelly JD (1982). Tissue levels of ascorbic acid during rat gestation. Int J Vitam Nutr Res.

[38] Kratzing CC, Kelly JD, Oelrichs BA (1982). Ascorbic acid in neural tissues. J Neurochem.

[39] Mahan DC, Ching S, Dabrowski K (2004). Developmental aspects and factors influencing the synthesis and status of ascorbic acid in the pig. Annu Rev Nutr.

[40] Harrison FE, Dawes SM, Meredith ME, Babaev VR, Li L, May JM (2010). Low vitamin C and increased oxidative stress and cell death in mice that lack the sodium-dependent vitamin C transporter SVCT2. Free Radic Biol Med.

[41] Huang-Doran I, Sleigh A, Rochford JJ, O'Rahilly S, Savage DB (2010). Lipodystrophy: metabolic insights from a rare disorder. J Endocrinol.

[42] Grundy SM (2015). Adipose tissue and metabolic syndrome: too much, too little or neither. Eur J Clin Invest.

[43] Campion J, Milagro FI, Fernandez D, Martinez JA (2006). Diferential gene expression and adiposity reduction induced by ascorbic acid supplementation in a cafeteria model of obesity. J Physiol Biochem.

[44] Abdel-Wahab YH, O'Harte FP, Mooney MH, Barnett CR, Flatt PR (2002). Vitamin C supplementation decreases insulin glycation and improves glucose homeostasis in obese hyperglycemic (ob/ob) mice. Metabolism.

[45] Kim B, Choi KM, Yim HS, Park HT, Yim JH, Lee MG (2018). Adipogenic and lipolytic effects of ascorbic acid in ovariectomized rats. Yonsei Med J.

[46] Han CY (2016). Roles of reactive oxygen species on insulin resistance in adipose tissue. Diabetes Metab J.

[47] Zatterale F, Longo M, Naderi J, Raciti GA, Desiderio A, Miele C, Beguinot F (2019). Chronic adipose tissue inflammation linking obesity to insulin resistance and type 2 diabetes. Front Physiol.

[48] Garcia-Diaz D, Campion J, Milagro FI, Martinez JA (2007). Adiposity dependent apelin gene expression: relationships with oxidative and inflammation markers. Mol Cell Biochem.

[49] Campion J, Milagro FI, Fernandez D, Martinez JA (2008). Vitamin C supplementation influences body fat mass and steroidogenesis-related genes when fed a high-fat diet. Int J Vitam Nutr Res.

[50] Garcia-Diaz DF, Lopez-Legarrea P, Quintero P, Martinez JA (2014). Vitamin C in the treatment and/or prevention of obesity. J Nutr Sci Vitaminol (Tokyo).

[51] Padayatty SJ, Levine M (2016). Vitamin C: the known and the unknown and goldilocks. Oral Dis.

[52] Levine M (1986). New concepts in the biology and biochemistry of ascorbic acid. N Engl J Med.

[53] Cheng CH, Kikuchi T, Chen YH, Sabbagha NG, Lee YC, Pan HJ, Chang C, Chen YT (2009). Mutations in the SLC2A10 gene cause arterial abnormalities in mice. Cardiovasc Res.

[54] Schneider CA, Rasband WS, Eliceiri KW (2012). NIH Image to ImageJ: 25 years of image analysis. Nat Methods.

