# Supplementary material for: Vitamin C attenuates predisposition to high-fat diet-induced metabolic dysregulation in GLUT10-deficient mouse model
Source: Genes Nutr. 2022 Jul 16;17:10. doi: 10.1186/s12263-022-00713-y (PMC9288715; doi:10.1186/s12263-022-00713-y)
Supplement: Supplementary file 1 — Additional file 1: Figure S1. Mice from different genotypes or treatment groups exhibit no significant differences in food intake, water intake, physical activity, or energy expenditure. Figure S2. AA supplementation has no obvious effect on histology of sWAT of HFD-fed GLUT10G128E and WT mice. [file 12263_2022_713_MOESM1_ESM.pdf]

# Supplementary figures

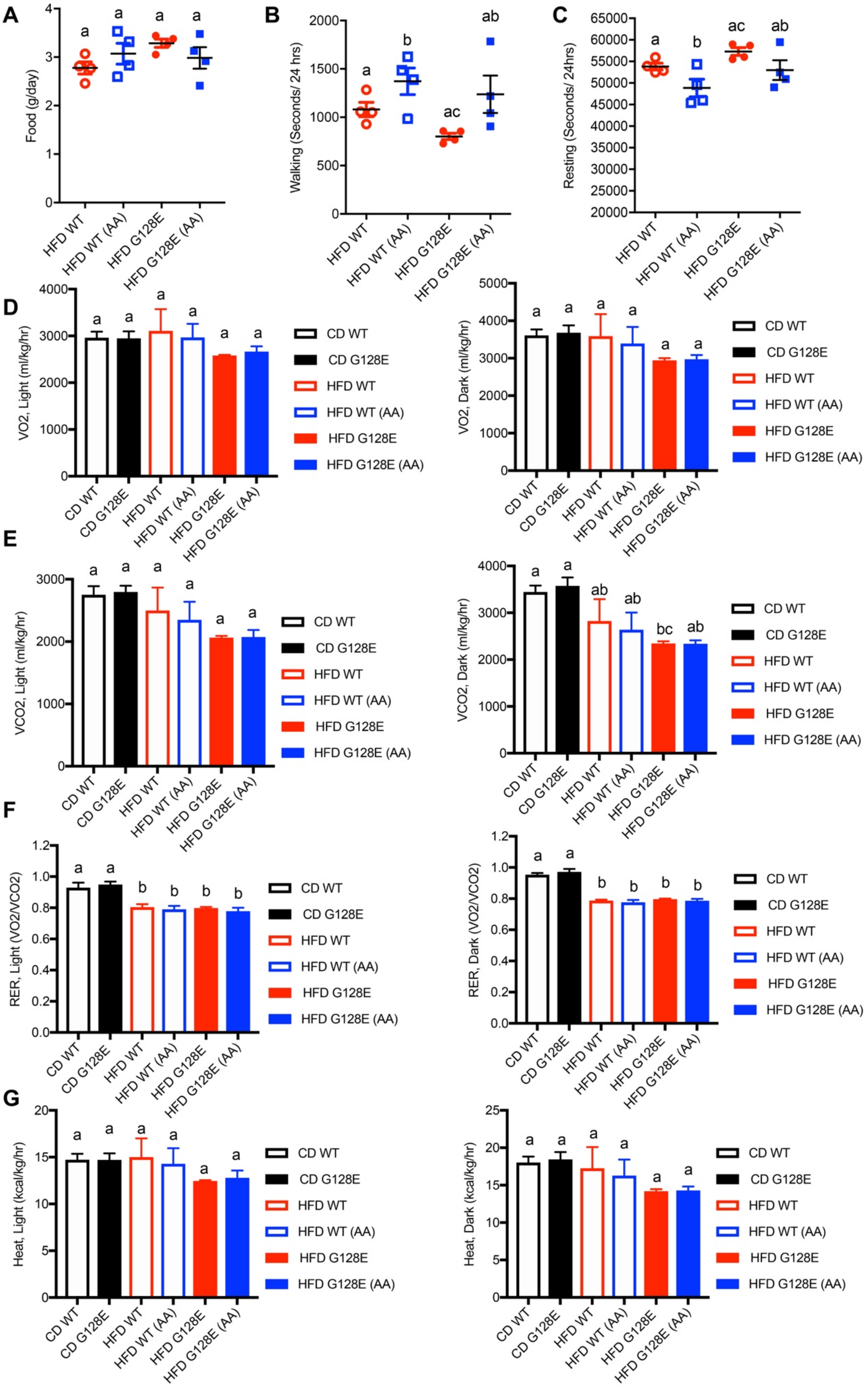

**Figure S1. Mice from different genotypes or treatment groups exhibit no significant differences in food intake, water intake, physical activity, or energy expenditure.** Mice were treated as described in Figure 1A. Data were collected from mice at the conclusion of feeding (20 weeks of age). (A) Average food intake over a 24-h period. (B) Walking and (C) resting time (seconds) over a 24-h period. Physical activity was measured in mice under a HFD using Clever Sys HomeCageScan TM3.0. (D–G) The metabolic indicators at 23℃ over a 79-h period. (D) Consumption of $O_2$, (E) $CO_2$ production, (F) Respiratory Exchange Ratio (RER = $VCO_2/VO_2$; an assessment of the metabolic exchange of oxygen for carbon dioxide), and (G) heat generation were measured by a comprehensive laboratory animal monitoring system (CLAMS). Bar graphs indicate the average values during the light (left panel) and dark (right panel) cycle; n = 4 mice in each group. The data are shown as mean ± SEM. Statistical significance was determined by one-way analysis of variance (ANOVA) followed by Tukey's test for multiple comparisons. *$P$ < 0.05, **$P$ < 0.01, ***$P$ < 0.001.

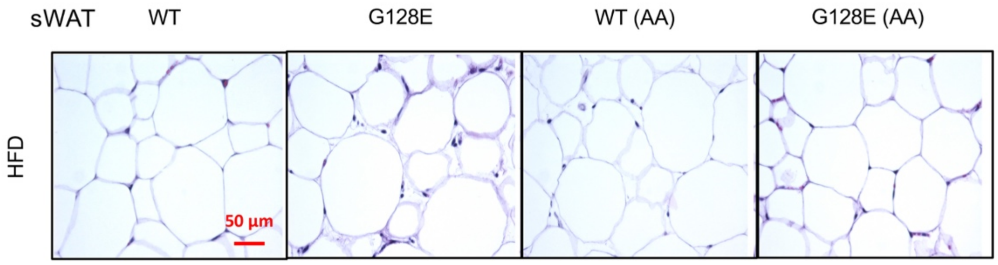


**Figure S2. AA supplementation has no obvious effect on histology of sWAT of HFD-fed *GLUT10*$^{G128E}$ and WT mice.** The sWAT sections were examined by H&E staining.
